# Characterization and Differentiation between Olive Varieties through Electrical Impedance Spectroscopy, Neural Networks and IoT

**DOI:** 10.3390/s20205932

**Published:** 2020-10-20

**Authors:** José Miguel Madueño Luna, Antonio Madueño Luna, Rafael E. Hidalgo Fernández

**Affiliations:** 1 Graphics Engineering Department, University of Seville, 41013 Seville, Spain; 2 Aerospace Engineering and Fluid Mechanical Department, University of Seville, 41013 Seville, Spain; antonio@madueno.es; 3 Graphics Engineering and Geomatics Department, University of Córdoba, 14014 Córdoba, Spain; ig1hifer@uco.es

**Keywords:** electrical impedance, SoC AD5933, artificial neural networks (ANNs), internet of things (IoT), temperature

## Abstract

Electrical impedance has shown itself to be useful in measuring the properties and characteristics of agri-food products: fruit quality, moisture content, the germination capacity in seeds or the frost-resistance of fruit. In the case of olives, it has been used to determine fat content and optimal harvest time. In this paper, a system based on the System on Chip (SoC) AD5933 running a 1024-point discrete Fourier transform (DFT) to return the impedance value as a magnitude and phase and which, working together with two ADG706 analog multiplexers and an external programmable clock based on a synthesized DDS in a FPGA XC3S250E-4VQG100C, allows for the impedance measurement in agri-food products with a frequency sweep from 1 Hz to 100 kHz. This paper demonstrates how electrical impedance is affected by the temperature both in freshly picked olives and in those processed in brine and provides a way to characterize cultivars by making use of only the electrical impedance, neural networks (NN) and the Internet of Things (IoT), allowing information to be collected from the olive samples analyzed both on farms and in factories.

## 1. Introduction

### 1.1. The Effect of Temperature

Temperature affects the texture of fruit and vegetables, both raw [1] and cooked [2], and it also affects their electrical parameters such as the electrical impedance of their pulp. A wide variety of procedures and equipment [3,4] exist to obtain various parameters associated with the pulp of these fruits and vegetables (penetration resistance, viscosity, etc.) 

In the case of olives, there are no studies explicitly analyzing the influence of temperature on the fruit, especially in its electrical parameters, although its effect is known in certain mechanized olive processing procedures. This is the case, for example, in the industrial olive pitting process [5], which is done by automatic machines where clamps are used to trap the olive, while a punch needle goes through the clamps and, consequently, the olives, causing them to be de-stoned. This type of machine reaches a considerable pitting speed, which obviously means a minimum cost per unit or fruit de-stoned. However, the very clamps that hold the fruit in place and the de-stoning tool cause breakage in a considerable number of olives during normal operation, which can be to the order of 14%, especially in the “Gordal Sevillana” olive variety. While the productivity of these pitting machines makes them more cost-effective despite the percentage of olive breakage compared to previous de-stoning systems, these machines present a serious problem, as the percentage of broken olives is quite considerable. 

While mechanical solutions reducing the percentage of olive breakage in such de-stoning machines do not exist, it has been proven that cooling the olives prior to pitting minimizes the problem to the point that, with adequate refrigeration, the percentage of broken olives during the pitting process is to the order of 2%, for the “Gordal Sevillana” olive variety previously cited. 

Furthermore, the current consumer trend for lower salt and acidity [6] means that a significant proportion of the olives deteriorate during the time they spend in fermenters. After a few months at an average temperature of 25 °C, the probabilities of having a significant loss of product in the pitting process are very high, due to a loss in firmness in the olives, especially the larger ones, which are the most in demand on the market. By cooling to temperatures close to 7 °C (in some cases it reaches 0 °C, depending on the cooling system and the costs the company wants to take on in the process), it is possible to increase the firmness of the olives while maintaining all of their characteristics, thereby reducing the loss of product due to the olives having a more rigid texture. In addition, this cuts down on the hours the machine is working, meaning a decrease in machine jams and hours when the punch needles are stopped.

One way of characterizing the state of the olives prior to de-stoning would be by doing an electrical impedance measurement on a representative sample. As will be demonstrated in this study, the electrical impedance of the olive pulp is not only affected by the olive variety or the type of industrial processing (green “Sevillana Style” or black “Californian Style” olives) [7], but also by the temperature. For this reason, characterizing this electrical parameter versus the temperature (especially between 7 °C and 0 °C) for a given type of industrial processing would show whether or not olives are apt before pitting.

### 1.2. Electrical Impedance: Measurement

The determination of electrical properties is used in a wide range of disciplines and industries [8]. In the agri-food sector, the use of electrical conductivity is applied for the determination of diverse characteristics in agri-food products [9,10] such as frost sensitivity, freezing tolerance, moisture content, and seed germination [11]. The use of electrical impedance spectroscopy is a technique that can provide very good results in the maturity stage of fruit and vegetables. In this paper, we are going to focus on a System on Chip (SoC) capable of a complex impedance measurement: the SoC AD5933 [12].

Impedance is a parameter of great importance to the characterization of circuits and electronic components [13], as well as the materials used in their production. Impedance (Z) is generally defined as the total opposition a device or circuit offers to the flow of an alternating current (AC) to a specific frequency, and it is represented as a complex number with a graphic representation on a complex plane. An impedance vector consists of the real part (resistance, R) and the imaginary part (reactance, X). Impedance can be expressed by using the rectangular coordinates in the form of R + j·X, or in the polar form as a magnitude and phase angle: |Z|∠Ø [14]. 

The instruments most commonly used to measure impedance are: the LCR meter or LCR bridge and the impedance analyzer. The first provides a simple and exact impedance measurement for a specific frequency value. However, for components other than pure inductors (L), capacitors ©, or resistors (R), it is inadequate to determine value. In these cases, an impedance analyzer is used to measure and graphically represent the complex impedance of the device being tested over a range of frequencies [15]. As they are high cost devices [16,17], the reason why designs using LCR meters (which are cheaper) exist, is to obtain a system impedance analyzer combining it with virtual instrumentation [12,18]. 

There are various configurations for the design of impedance measuring bridges such as Schering [19] and the Maxwell bridge [20]. The difficulty with these is that they need to have the balance condition. Moreover, they are generally used for pure inductive or capacitive impedance measurements. In order to obtain the complex impedance, electronic methods are used, such as the vectoral method and the method using two quadrature sinusoidal waves [21,22].Another way to measure the complex impedance is the three-voltage method [23]; however, this requires voltages to be raised to the square, which makes the measurement errors greater, in addition to the fact that it requires very precise instruments to run the measurements. 

An Impedance/Gain-Phase analyzer [24] is a measuring instrument of great value for the study and design of electronic circuits. This powerful device is capable of obtaining diagrams separately, both in magnitude and in phase of any network or electronic circuit which has an input and an output. With this information, it is possible, for example, to obtain the transfer function of a specific circuit, although its implementation is not known. 

An Impedance/Gain-Phase analyzer should be capable of obtaining a reliable amplitude and phase diagrams corresponding to a specific circuit. In both cases, these variables will be represented according to frequency. These types of analyzers must be capable of generating a sinusoidal signal and directly applying it to the input of the network to be measured. Thus, a frequency sweep is done on the network with a specific criterion regarding the initial, intermediate, final frequency values, number of points, linear or logarithmic sweep, etc., which is normally selected by the user. To generate the magnitude diagram, it is necessary to find the quotient between the amplitudes of the network output and input for each of the values to be measured in the frequency sweep. Furthermore, in order to do the phase diagram, it is necessary to obtain the phase difference between the network output and input signals again for each of the frequency values. Therefore, it is obvious that in order to build an impedance/gain-phase analyzer, a sine wave generator is necessary, at the very least, to generate a circuit frequency sweep capable of measuring amplitude and of another circuit capable of measuring the phase difference between two signals. 

### 1.3. Previous Examples of the Use of Electrical Impedance in Olive Production and Other Fruits

Electric conductivity has been used (only its magnitude) to characterize different olive varieties (*Olea europa* L.) [25], specifically, four different cultivars: “Picual”, “Manzanilla de Sevilla”, “Hojiblanca” and “Gordal Sevillana” with the objective of establishing a fruit maturity index and, thus of determining the optimal time for harvesting based on parameters such as oil yield and quality, demonstrating that the conductivity increased with fruit maturity and that each variety had an average characteristic electric conductivity value in the last stages of maturation. Previous studies have focused in particular on determining maturity indexes and quality parameters [7,26,27], or on ways of slowing the maturation period, above all in climacteric fruits, with the aim of lengthening the period between harvesting and commercial consumption [28].

### 1.4. Neural Networks (NN) for Adjustment and Sorting

Artificial neural networks are computational models inspired by the behavior observed in their biological counterparts [29]. Their use is widespread in an abundance of fields. Focusing on the table olive, we can see their use in [30] or in [31].

In this study, we are going to use two types of neural network. On the one hand, a neural network for adjustment [32] to evaluate the effectiveness of this technique in generating a valid impedance behavior mode for olive pulp and, on the other hand, a neural network for sorting [33] to distinguish between olive varieties at different temperatures. 

### 1.5. The Internet of Things (IoT) 

The use of the IoT is widely extended nowadays and there are numerous cases (for example [34,35,36,37,38]) in precision agriculture, irrigation, temperature control, monitoring of the agricultural production process, in automated olive chain processes, or in the operation of the pitting, slicing, and stuffing machines themselves (DRR) [30,31]. This paper shows the development of equipment based on the SoC AD5933 [12] which, together with a neural network and an IoT system, allows for the analysis of the state of the olives on the farm or in the factory before pitting.

The general objective set out by this paper is to develop an electrical impedance measurement system adapted to table olives, making use of the SoC AD5933. In order to do this, two tasks are going to be done:

(1) Impedance modeling through neural networks for two varieties of olives (“Gordal Sevillana” and “Hojiblanca”), cured in caustic soda and fermented in brine (an industrial process known as “Estilo Sevillano”).
(2) Classification via neural networks for each olive variety at three temperatures (25 °C, 7 °C, and 0 °C).

To this end, these systems will be developed:

- A specific device with the SoC AD5933 [12], that includes an I^2^C communication interface [39], an external DDS generator based on a FPGA XC3S250E-4VQG100C [40] to conduct a complete sweep from 1 Hz to 100 kHz and two ADG706 analog multiplexers [41] to set the impedance range to be measured. This device is controlled by a 32-bit ARM CORTEX M3 AT91SAM 3 × 8 E microcontroller working at 84 MHz [42].
- The system control software using Matlab programing language [43].
- IoT communication is based on [44] to generate a database with the trial results.

## 2. Materials and Methods

### 2.1. Olive Varieties and Industrial Process Used

The olive fruit (*Olea europaea* L.) [25] is an ovoid drupe whose size oscillates from 0.6 to 2 cm in diameter and from 1 to 4 cm in length. This size depends on the variety (see Figure 1 in [25]), the vegetative state of the tree, the environmental conditions, and the cultivation techniques [45]. We used samples of 100 unripe olives from olive orchards (200 trees ha^−1^, Seville, Spain), under irrigation and non-limiting nutrient conditions with mechanical harvesting, and samples of 100 olives in brine from a factory in Seville. In this work we used two varieties: “Hojiblanca” and “Gordal Sevillana” freshly picked in class 0, 1, and 2 [46] and processed in brine the “Estilo Sevillano” way. This type of olive, seasoned this way, is processed in four stages [47,48,49]:

- Lye treatment in NaOH 2–4% (p/v) for 6–12 h
- Rinsed in water (12–15 h)
- Fermented in brine (10–12% (p/v) for 60–300 days)
- Pitted and stuffed or sliced.

### 2.2. The SoC AD5933 to Measure Impedance

According to the SoC AD5933 datasheet [12], “the AD5933 is a high precision impedance converter combining an integrated frequency generator with a 12-bit and 1 MSPS analogue-to-digital converter (ADC)”. The impedance of the device under test (DUT) is sampled by the internal ADC and is processed with a discrete Fourier transform (DFT)” [50]. The output excitation voltage and the measurement frequency are totally programmable. Communication is done via an I2C interface. 

Reviewing the available bibliography confirms that the SoC AD5933 has significant biological applications, such that it has been used to monitor the growth of cell cultures [51,52] in measurements in isolated cells [53], detection of blood clotting [54], biosensor applications [55], and bio-impedance measurements [56,57,58,59,60]. Likewise, it is used in the monitoring of “technical objects”, for example, for corrosion analysis in steel structures [61,62]. In order to obtain comprehensive information about the electrical properties of a measured object and to use a suitable impedance spectrum analysis method (equivalent circuit modelling), impedance must be measured at a wide range of frequencies [63]. 

In Table 1, the technical data of various impedance meters based on the SoC AD5933 are shown. Only three of the devices described allow impedance were to be measured in more than three orders of magnitude of frequency. The impedance range measured is typically from 10 Ω to more than 10 MΩ. However, in many cases, it does not give the exact range. The majority of the impedance meters mentioned require additional analog front-ends to provide an adequate interface between the SoC AD5933 and the device under test (DUT) [64]. 

Other authors [65,66,67] proposed modifications in the original topology provided by the manufacturer with the use of a multiplex system to adjust the range of the impedance measurement, although this does not include changes in the source clock input into the SoC AD5933. In [68] the operation of the SoC AD5933, the hardware and software developed, and the IoT system are described.

### 2.3. Neural Networks to Modeling and Sorting

Two types of neural networks were used from the Matlab libraries:

- A neural network for adjustment, “fitnet” [32], allows a description of the evolution of the complex impedance in the pulp of 2 varieties of olives to be obtained. It is a considerable improvement over the Hayden model [69].
- A sorting network, “patternnet” [33], to distinguish between 6 cases (2 varieties and 3 temperatures) in unripe and another 6 cases (2 varieties and 3 temperatures) in olives processed the “Estilo Sevillano” way.

## 3. Results

Three verification tests of the developed hardware have been carried out (Section 3.1, Section 3.2 and Section 3.3) as well as a study on the effect of variety on electrical impedance in unripe olives (Section 3.4) and on olives in brine (Section 3.5). A model with neural networks on the evolution of impedance in unripe and brined olives of the two varieties studied in Section 3.6. and a classifier based on neural networks capable of distinguishing between 6 different cases in unripe olives was trained (Section 3.7.1), in brined olives (Section 3.7.2), and finally (Section 3.8) the IoT system used is shown.

### 3.1. Impedance Meter Verification Testing: DUT Made Up of a Pure Resistance of 10 kΩ

**Figure 1:**
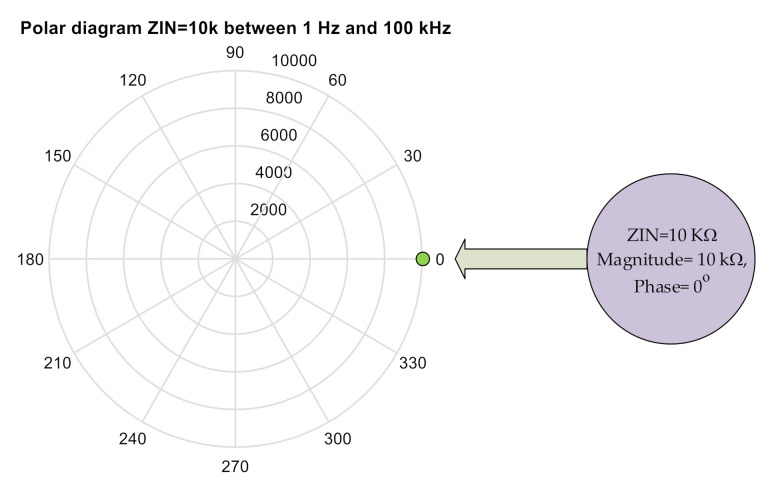
Polar diagram: Complete sweep from 1 Hz to 100 kHz with ZIN = 10 KΩ (Magnitude 10 kΩ, Phase 0°).

A test has been done using a pure resistance of 10 KΩ, the results are in Figure 1. The object of the test is to make sure that the equipment is working correctly. As can be seen, the answer is the same in both the magnitudes as in the phase throughout the sweep from 1 Hz to 100 kHz. 

### 3.2. Impedance Meter Verification Testing: DUT Comprised of a SERIAL RLC Circuit 

We conducted a test on the SERIAL RLC circuit with an inductance of L = 56 mH, C = 1500 pF and R = 5.1 kΩ, with Zcal = 10 k, ZBF = 10 k, Range 1: 2 Vpp, PGA = ×1, Multiplier = ×1 from 1 Hz to 100 kHz (see Tables 1 and 2 in [68]).

For the test, three elements were connected in series to build a typical RLC circuit. The theoretical resonance frequency is:

(1) $$f_{o\;}=\;\frac{1}{2\cdot \pi \cdot \sqrt{L\cdot C}}\;=\;\frac{1}{2\cdot \pi \cdot \sqrt{{56\;\times \;10}^{-3}{\;\times \;1500\;\times \;10}^{-12}}}\;=\;17365.22\;\mathrm{Hz}$$

**Figure 2:**
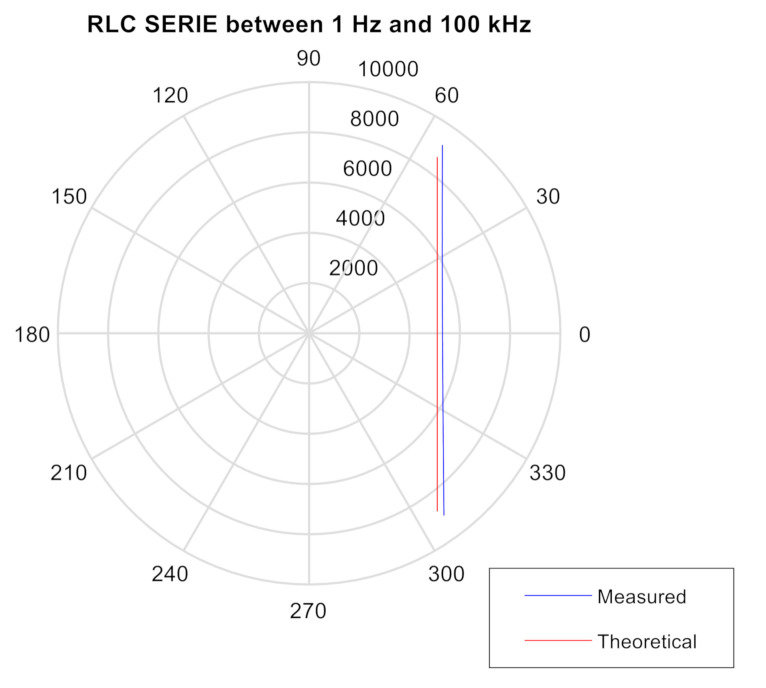
Polar diagram of the test with SERIAL RLC circuit between 1 Hz and 100 KHz. The blue values are measured and the red ones are theoretical.

A low frequency predominates the capacitive part with negative phases that tend to −90°. In the proximity of the resonance zone, the phase tended to zero and from that moment the inductive part began to predominate, the phase in that case positive tends to 90°. The theoretical and experimental results are shown in Figure 2. The small discrepancies between theoretical and experimental data are due to the tolerance of the components used in the real test.

### 3.3. Impedance Meter Verification Testing: DUT Comprised of a PARALLEL RLC Circuit

The test on the PARALLEL RLC circuit with an inductance of L = 56 mH, C = 1500 pF and R = 5.1 kΩ, with Zcal = 10 k, ZBF = 10 k, Range 1: 2 Vpp, PGA = ×1, Multiplier = ×1 from 1 Hz to 100 kHz (see Tables 1 and 2 in [68]).

For this test, three elements have been connected in parallel to build a typical RLC circuit in parallel. The theoretical frequency will be the same as in the previous test.

**Figure 3:**
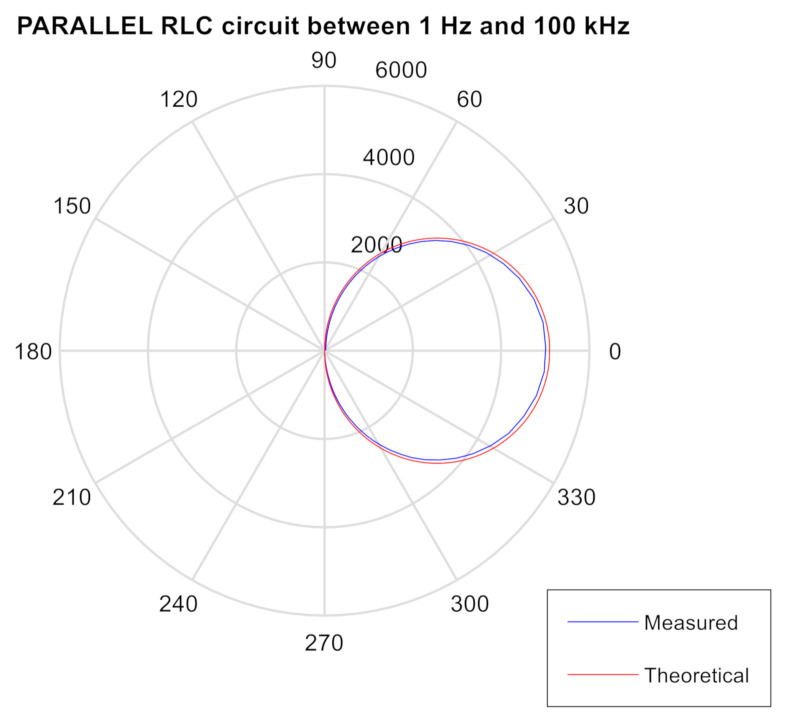
Polar diagram of the test with the PARALLEL RLC circuit between 1 Hz and 100 kHz. The blue values are measured and the red ones are theoretical.

The theoretical and experimental results are shown in Figure 3. Once again, the differences are due to the tolerance of the components used.

### 3.4. Test on Unripe Olives: The Effect of Olive Variety on Electric Impedance

**Figure 4:**
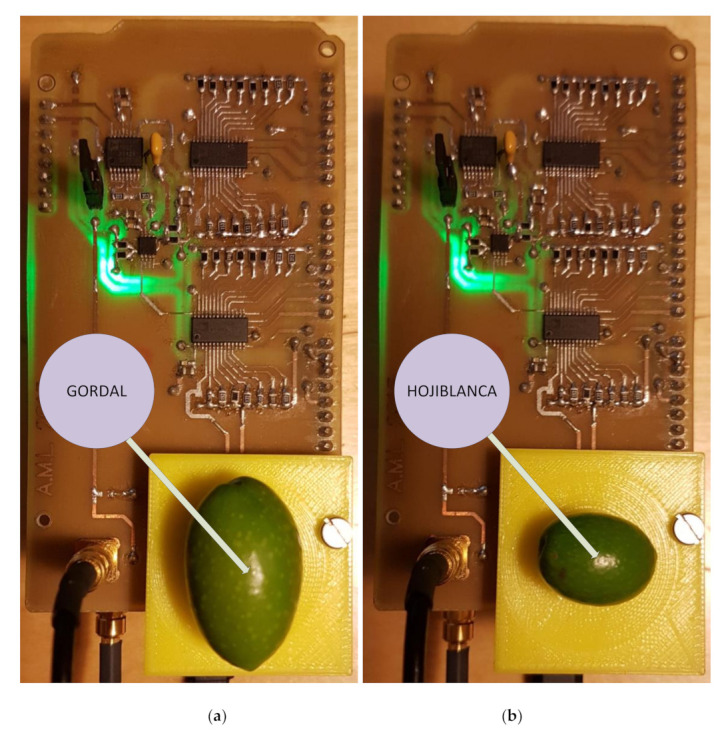
(**a**) “Gordal Sevillana” and (**b**) “Hojiblanca” olive varieties during the electrical impedance measurement.

This study has dealt with olive samples from the “Gordal Sevillana” and “Hojiblanca” varieties, shown in Figure 4, from two olive trees situated side by side and therefore subject to the same environmental and watering conditions. These samples were harvested on the same day at the same time (16/06/2019).

**Figure 5:**
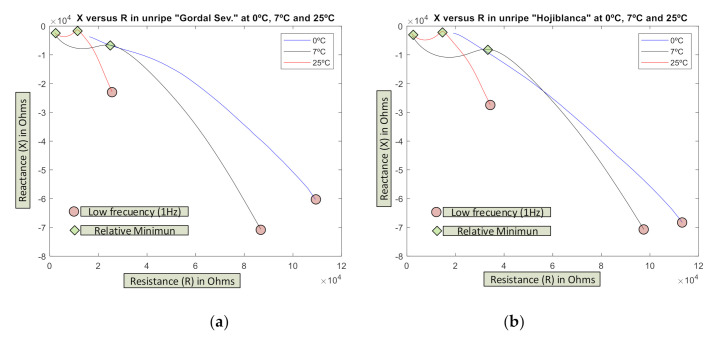
Evolution of the impedance in (**a**) unripe “Gordal Sevillana” and (**b**) “Hojiblanca” varieties at 3 temperatures: 0 °C, 7 °C, and 25 °C (average of 100 tests). The red circles corresponds to f = 1 Hz.

In Figure 5, the evolution of the impedance profile obtained (an average of 100 tests) appears presented as its components X-R for unripe olives of both varieties at three temperatures: 0 °C (blue), 7 °C (yellow), and 25 °C (red).

As can be seen, they present a characteristic profile differentiated both by temperature and maximum values, the “Hojiblanca” being the one with higher values in both components X, R, and thereby the module Z, both at 0 °C and 7 °C, while at 25 °C its profile is similar to the “Gordal Sevillana”.

Previous tests have demonstrated that these impedance profiles do not adjust well to models of the type described [70] (see Figure 3b of said citation) with minimum at X close to zero. Conversely, in unripe olives at a low frequency, there are high values in both R and X (red circles on the graphs). Likewise, two relative minimums can be observed in the reactance X value (green diamonds) at 7 °C and 25 °C. The trend on the graph of 0 °C suggests that at frequencies over 100 kHz it would also exist in this case.

One of the problems that sometimes appears is distinguishing between the unripe varieties, due to the fact that their outward appearance and size cause doubt. This test demonstrated how these varieties (unripe) affect the electrical impedance the same as the rest of the parameters (especially temperature). This system would allow the sample variety of olive to be identified from the electrical impedance without using other characterized parameters.

**Figure 6:**
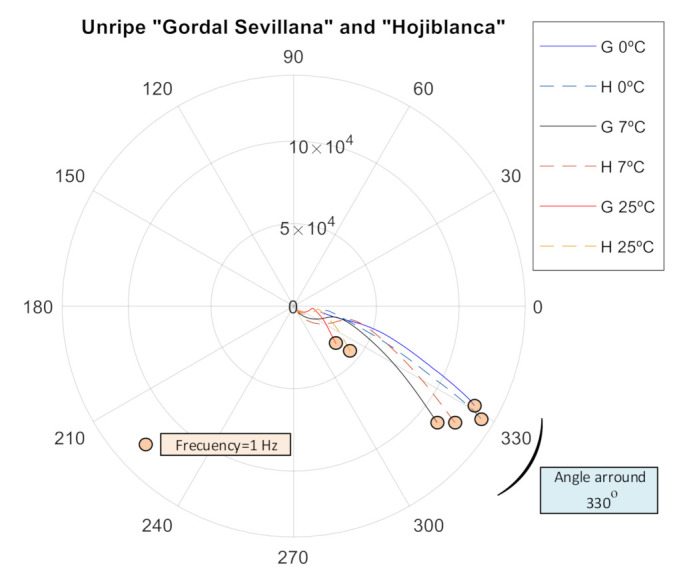
Frequency sweep to measure the electrical impedance of the “Gordal Sevillana” and “Hojiblanca” varieties when unripe (average values from 100 tests).

In Figure 6, the impedance spectrum of both varieties is shown together (in polar diagram). Each one has a characteristic profile at each temperature and the maximum difference is found at low frequencies, which could serve to distinguish between unripe olive varieties as is also indicated in [25].

### 3.5. Test on Processed Olives: The effect the Olive Variety Has on Electrical Impedance in Olives Processed the “Estilo Sevillano” Way 

Just as with unripe olives, it is sometimes difficult to distinguish between processed varieties because of their shape, size, and special color (due to the chemical processes they undergo) and they are very similar. Once again, a study was done on the two previously mentioned varieties (“Gordal Sevillana” and “Hojiblanca”) seasoned the “Estilo Sevillano” way.

**Figure 7:**
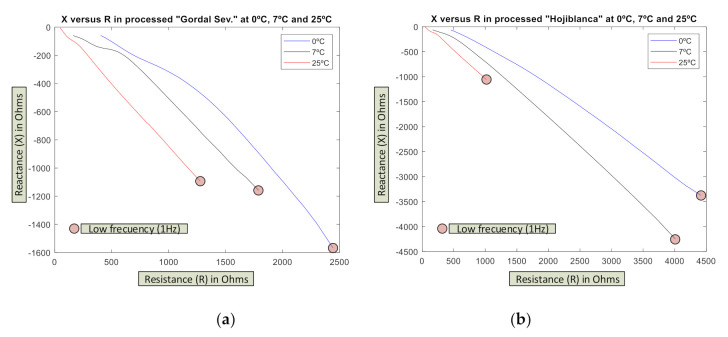
The effect of temperature on electric impedance on the (**a**) “Gordal Sevillana” and (**b**) “Hojiblanca” olive varieties processed in the “Estilo Sevillano” way in at 3 temperatures: 0 °C, 7 °C, and 25 °C (average values of 100 tests).

In Figure 7, the effect of temperature is shown on these types of olive with 100 samples of each variety at 3 temperatures (0 °C, 7 °C, and 25 °C). Once again, each variety has an electrical impedance spectrum characteristic at each temperature and the maximum differences between the two varieties are at lower frequencies.

It must be highlighted that in the case of R-X curves, minimums relative to X do not present as happened in the case of unripe olives. The classic models described in [69,70] are not applied. 

Another detail to emphasize is the drastic reduction of the values in both components (R, X) (around 20 times) due to the presence of brine, which makes the olive pulp highly conductive. 

**Figure 8:**
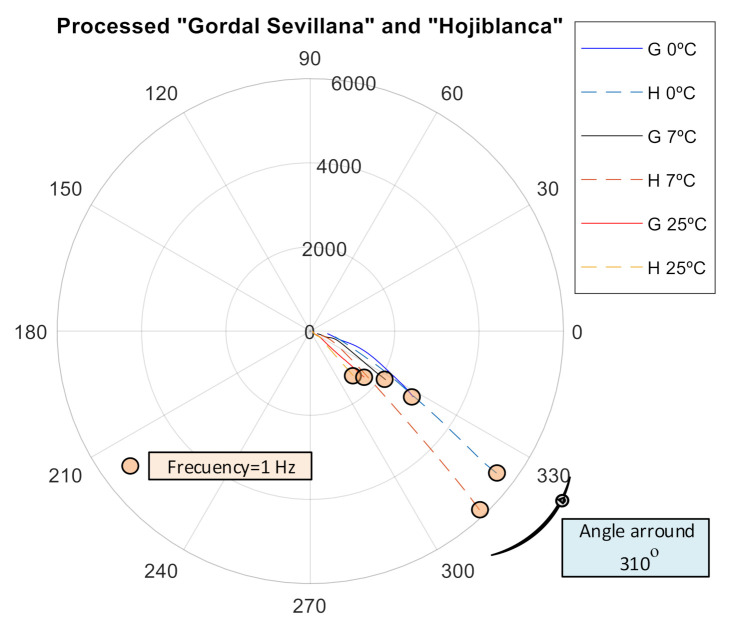
Influence of the brine on the impedance of processed olives.

Finally, as can be observed, comparing Figure 6, Figure 7 and Figure 8, the presence of brine makes the aggregate behave in a more capacitive way with an average offset angle around 310° for olives in brine and around 330° for unripe olives.

### 3.6. Complex Impedance Model for “Gordal Sevillana” and “Hojiblanca” Olive Varieties Unripe and Processed the “Estilo Sevillano” Way via Neural Networks 

As mentioned in Section 2.3, the classic models [69,70] do not adapt well to characterize the evolution of impedance in olives; not in unripe olives or in those processed the “Estilo Sevillano” way, for each variety and each temperature. Thus, the Hayden model [69] does not take components R2 and C3 sufficiently into account, reducing their effect.

**Figure 9:**
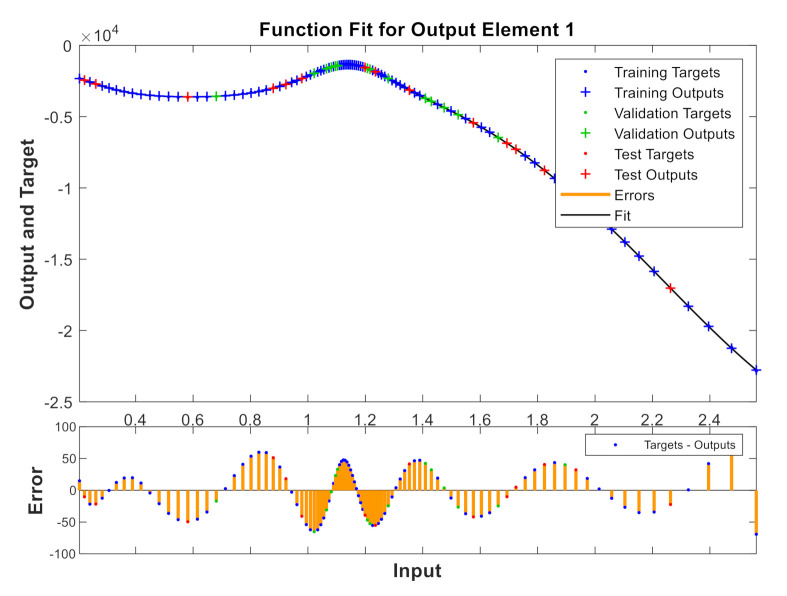
Unripe “Gordal Sevillana” at 25 °C.

**Figure 10:**
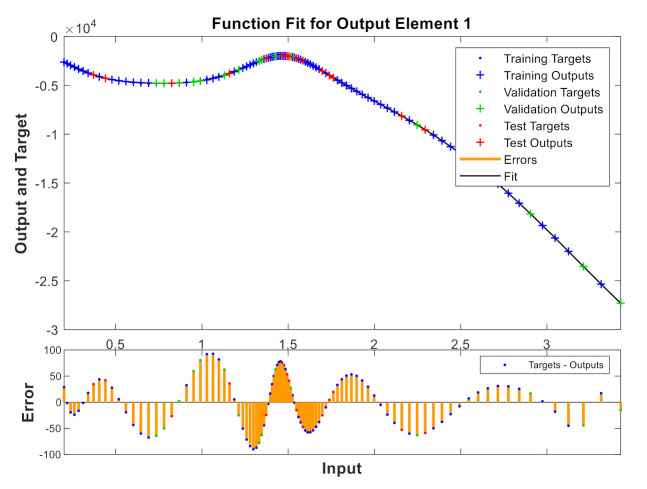
Unripe “Hojiblanca” at 25 °C.

**Figure 11:**
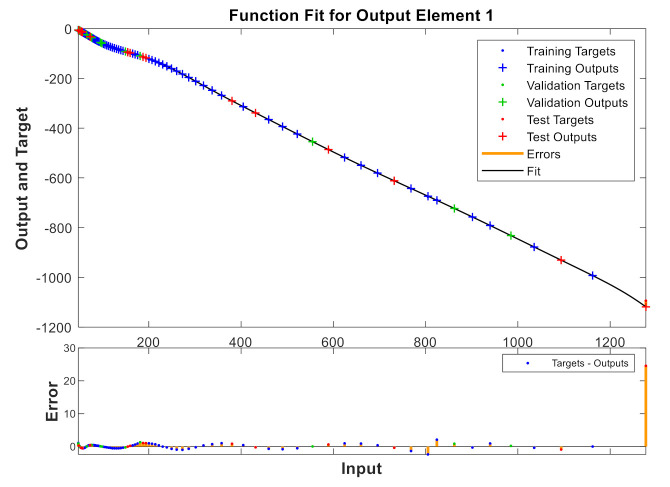
Processed “Gordal Sevillana” at 25 °C.

**Figure 12:**
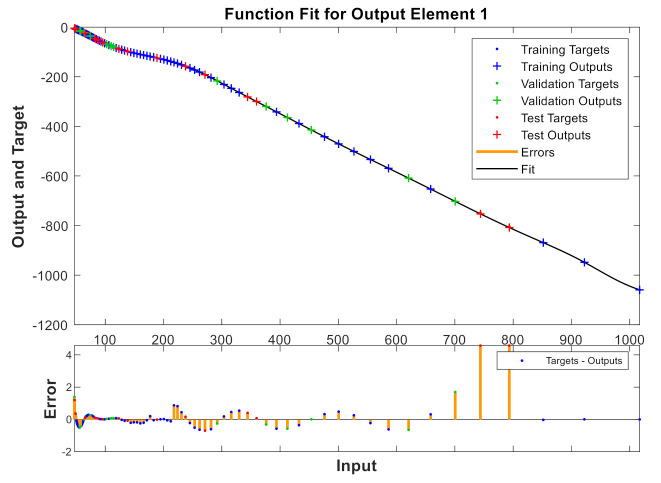
Processed “Hojiblanca” at 25 °C.

A model has been calibrated based on neural networks for both varieties, both in unripe and in processed olives at the three temperatures previously seen and directly correlating to the values of the reactive X and resistive R components. Figure 9, Figure 10, Figure 11 and Figure 12 show some results using the Matlab Toolbox neural networks and its fitnet function [32], with a hidden layer hiding 5 neurons being enough to obtain the adequate adjustment.

The goodness of fit is expressed in agreement to [71] and as relative error in % in Table 2 and Table 3.

### 3.7. Sorting with Neural Networks According to Variety and Temperature 

Distinguishing between varieties, either unripe or processed (in this case, “Estilo Sevillano”), can be of interest as mentioned in Section 2.3 to differentiate between varieties in case of doubt. In this section, the sorting is done using Toolbox by Matlab and, specifically, its neural sorter patternnet [33]. In all cases, adjustments were made by using just one layer of 8 neurons, 2 varieties, and 3 temperatures, in order for the sorter to distinguish between 6 possible options. Conducting the sorting process at 3 temperatures allows it to be generalized given that the temperature conditions on the farm and in the factory can be different throughout the work day or the time of year when the test is done. This is of special interest in the industry where olives are generally found at these three temperatures (room temperature during storage is about 25 °C on average, at 7 °C prior to pitting/stuffing/slicing and 0 °C during the pitting/stuffing/slicing).

#### 3.7.1. Sorting with Neural Networks for Unripe Olives

**Figure 13:**
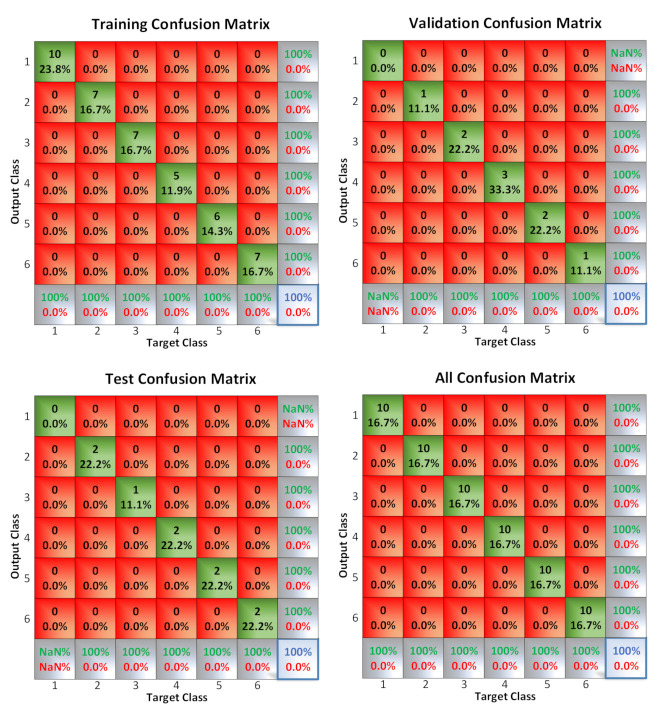
Training confusion matrix for the sorting of unripe olives.

Once the sorter is trained (10 samples of each state and 5 challenges, which is to say 60 samples all together for training and 30 to test the sorter), the results are shown in Figure 13. The neural network detected 100% of cases correctly.

#### 3.7.2. Sorting with Neural Networks for “Estilo Sevillano” Processed Olives 

**Figure 14:**
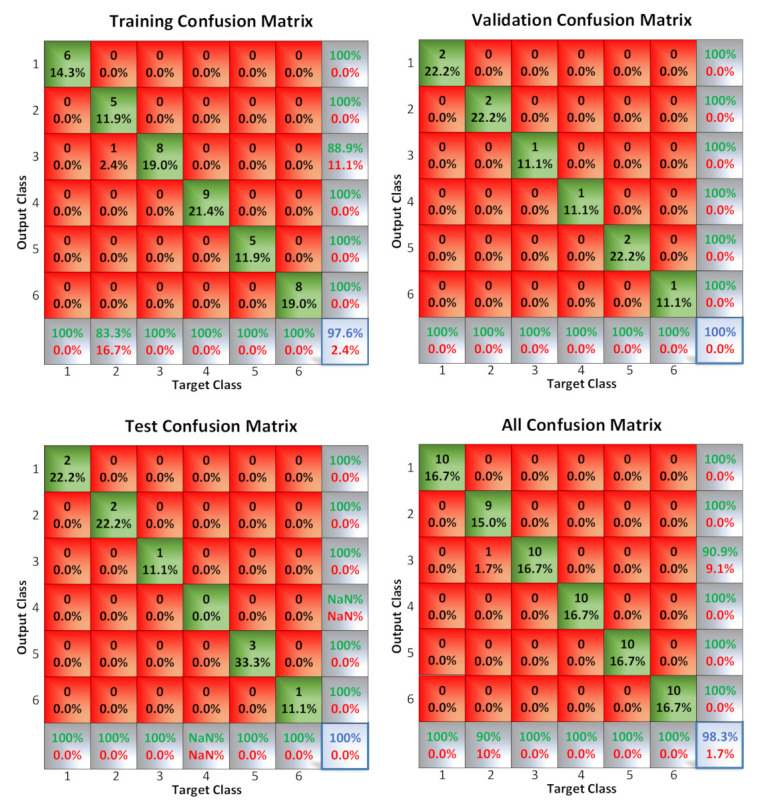
Training confusion matrix for sorting “Estilo Sevillano” processed olives.

Once the sorter is trained (10 samples of each state and 5 challenges, which is to say 60 samples all together for training and 30 to test the sorter), the results are shown in Figure 14. The neural network detected more than 98% of cases correctly.

### 3.8. The Internet of Things (IoT)

**Figure 15:**
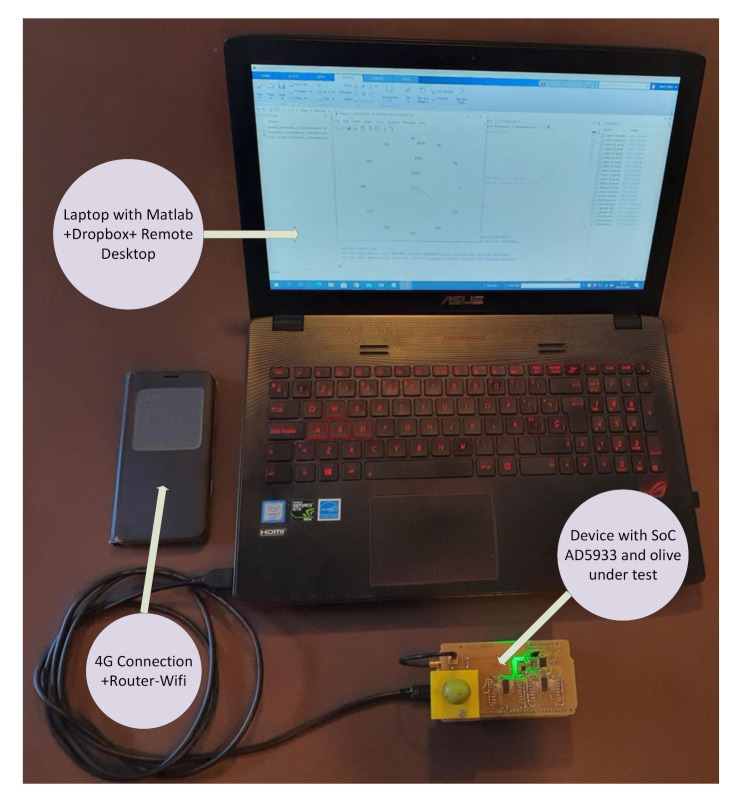
IoT System during test in factory.

With the objective of evaluating the prototype for the farm and factory tests, a simple IoT system was chosen based on the use of a laptop which executes the Matlab application and sends the file with the results to a shared folder in a Dropbox. This way, the samples can be analyzed remotely and from any other computer, as shown in Figure 15.

## 4. Conclusions

With this work it has been possible to verify that:

- Both unripe and processed olives present an impedance profile equivalent to an R-C model without an inductive component with a phase around 330° in unripe olives and 310° in brined olives.
- With unripe and processed olives, a characteristic impedance profile can be observed for each variety at each temperature. It is at a low frequency where the differences are more accentuated.
- With unripe olives at a high frequency, the relative minimums observed are similar to those described by classic models like that of Hayden, which does not happen with olives processed in brine.
- With olives processed in brine, the impedance value of the components R and X are reduced by 20 times, due to the effect of the brine.
- The models developed with neural networks like fitnet to represent the evolution of the impedance allow a model of type R versus X be obtained with only 5 neurons in the hidden layer.
- It has been verified that neural networks like patternnet and 8 neurons in the hidden layer, allow it to distinguish between the 6 cases studied (2 varieties and 3 temperatures) both in unripe and processed olives.
- The use of a simple IoT system based in Dropbox allows samples to be obtained on the farm and in the factory for later study using the combination of a laptop with a 4G connection and the prototype developed.

On the other hand, to reach these results, the design of a hardware has been chosen that has allowed us to obtain the maximum benefits of the SoC AD5933:

- A circuit implementing an SoC AD5933 has been developed with all the peripheral elements necessary for it to run. This prototype includes a pair of ADG706 analog multiplexers in order to convert the range in the impedance module to be measured.
- In order to achieve the maximum resolution in the DFT, a DDS based on an FPGA has been used to generate a clock signal to be programmed at will according to the limits of the frequency sweep to be carried out during the impedance measurement.
- Programming the main application has been done in Matlab. In order to control all of the elements, an ARM CORTEX M3 (AT91SAM3X8E) microcontroller has been used with an Arduino DUE, implementing all of the firmware necessary to control the hardware. Lastly, the Picoblaze routine control embedded in the FPGA of the DDS has been implemented in ASM.
- The resistance tests and the RLC series/parallel circuits have shown that the system works properly.
- There is a functional limitation to the chip where the internal DSP speed is proportional to the clock speed applied externally. In these circumstances, for the low frequency measurement (from 1 Hz to 30 Hz), a 25 kHz clock has been used which, compared to the frequency used (16 MHz), makes the measurement process 640 times slower at low frequencies.
- The circuit built is experimental and, in order to use it directly on the farms where the crop is located, a version capable of withstanding those working conditions ought to be produced. Likewise, the application should be an app, for example on a cell phone or a tablet, where it could connect to the computer via Bluetooth.
- One possible option is to implement all the routines through a microcontroller embedded in the FPGA, using a programable microcontroller directly in C (for example, Microblaze) in this case.
- Finally, among the future areas of work, we are considering:
- Increasing the frequency range to 25 MHz (likely on a system which allows it to go over 100 kHz) with the objective of seeing if, after that point, they are standard application models like in Hayden’s.
- Studying other olive varieties of commercial relevance like “Manzanilla” or “Cacereña” olives.
- Studying other industrial treatments like the oxidized black olive (California style).
- Running an analysis which correlates the breakage percentage in DRR machines directly with the measured impedance value of different varieties, processes, and temperatures.

Future studies will include the application of this methodology in other fruits such as tomato or cherry.

## Figures and Tables

**Table 1:** List of electrical impedance meters based on the SoC AD5933 (taken from [64]).

Author	Purpose	Frequency Range	Impedance Range	Maximum Error
C. J. Chen et al. [51]	Monitoring Cell Cultures	Set to 10 Hz	Not specified	Not specified
T. Schwarzenberger et al. [52]	Monitoring Cell Cultures	100 Hz–100 kHz	Not specified	2%–magnitude, 2%–argument
M. H. Wang et al. [53] (uses an AD5934)	Measuring Isolated Cells	0.1 Hz–100 kHz	100 Ω–10 MΩ	Around 10% for cell measurement
J. Broeders et al. [55]	Biosensor Application	10 Hz–100 kHz	10 Ω–5 MΩ	Not specified
P. Bogónez-Franco et al. [57]	Bioimpedance Monitor	100 Hz–200 kHz	10 Ω–1 kΩ	2.5%–magnitude, 4.5%–argument
J. Ferreira et al. [58]	Bioimpedance Electrodes In Clothing	5 kHz–450 kHz	Not specified	0.7%–resistance, 17%–reactance
C. Margo et al. [59]	“Embedded” applications for bioimpedance	1 kHz–100 kHz	Not specified. No data	2.5%–magnitude, 1.3%–argument
A. Melwin y K. Rajasekaran [60]	Body composition measurements	Set to 50 kHz	Not specified	2% (not specified)
J. Hoja y G. Lentka [61,62]	Object monitoring technique	0.01 Hz–100 kHz	10 Ω–10 GΩ	1.6%–magnitude, 0.6%–argument

**Table 2:** Goodness of fit for unripe olives according to variety and temperature.

Variety	Temperature	P	Relative Error (%)
	0 °C	7.3753 × 10^3^	1.33%
“Gordal Sev.”	7 °C	788.9215	0.08%
	25 °C	1.2965 × 10^3^	0.33%
	0 °C	1.0014 × 10^3^	0.18%
“Hojiblanca”	5 °C	294.0392	0.05%
	20 °C	2.1827 × 10^3^	0.36%

**Table 3:** Goodness of fit for processed olives according to variety and temperature.

Variety	Temperature	P	Relative Error (%)
	0 °C	1.6073 × 10^3^	1.14%
“Gordal Sev.”	7 °C	1.885 × 10^3^	0.34%
	25 °C	6.3865	1.27%
	0 °C	1.4252 × 10^3^	0.25%
“Hojiblanca”	7 °C	6.321 × 10^3^	1.14%
	25 °C	2.218 × 10^3^	0.4%
